# Supplementary material for: Balancing time use for children’s fitness and adiposity: Evidence to inform 24-hour guidelines for sleep, sedentary time and physical activity
Source: PLoS One. 2021 Jan 19;16(1):e0245501. doi: 10.1371/journal.pone.0245501 (PMC7815105; doi:10.1371/journal.pone.0245501)
Supplement: S1 File — Analytical method. (PDF) [file pone.0245501.s001.pdf]

S1 File. Balancing time use for fitness and adiposity: evidence to inform 24-hour guidelines

Analytical method

1. Models

Regression models were used to explore the relationship between time-use composition and the outcome. To avoid perfect multi-collinearity and enable the time-use composition (% of daily time spent in sleep, sedentary time, light physical activity (LPA) and moderate-to-vigorous physical activity (MVPA)) to be included in the model, the percentages of time were expressed as isometric log ratios (*ilrs*). The default *ilr* transformation from the R Compositions package was used. For the purpose of this paper, the type of *ilr* transformation is irrelevant, as we do not seek to interpret individual *ilr* regression coefficients. The ensuing analyses always involve the whole set of *ilrs*, together. Taken together, the set of *ilrs* contains the same relative information regardless of the type of *ilr* transformation used.

Quadratic terms were tested for the set of *ilrs*, to see if the relationship between time-use composition and outcomes was non-linear. The quadratic terms were retained if they improved the model fit ($p<0.05$) according to the partial F test for nested models.

The following code fragment shows that quadratic terms were not indicated for the adiposity composite outcome ($p=0.15$).

```
> mod4=lmrob(adiposity~ilr(comp)+ ses+ sex +age + puberty,control=a1)
> car::Anova(mod4, test.statistic="F")
Analysis of Deviance Table (Type II tests)

Response: adiposity
            Df       F    Pr(>F)
ilr(comp)    3 14.9267 1.535e-09 ***
ses          1 12.3526 0.0004571 ***
sex          1  9.6872 0.0019004 **
age          1 20.0506 8.277e-06 ***
puberty      1 15.8272 7.365e-05 ***
Residuals 1174
---
Signif. codes:  0 ‘***’ 0.001 ‘**’ 0.01 ‘*’ 0.05 ‘.’ 0.1 ‘ ’ 1
```

```
> modsq=lmrob(adiposity~ilr(comp)+sq(ilr(comp))+ ses+ sex +age + puberty,control=a1)
> car::Anova(modsq, test.statistic="F")
Analysis of Deviance Table (Type II tests)

Response: adiposity
                Df       F    Pr(>F)
ilr(comp)        3  3.2875 0.0201098 *
sq(ilr(comp))    6  1.5582 0.1559545
ses              1 12.1721 0.0005031 ***
sex              1  9.4497 0.0021609 **
age              1 19.6302 1.028e-05 ***
puberty          1 15.2919 9.742e-05 ***
Residuals     1168
---
Signif. codes:  0 ‘***’ 0.001 ‘**’ 0.01 ‘*’ 0.05 ‘.’ 0.1 ‘ ’ 1
```

```
> anova(mod4, modsq)
Robust Wald Test Table

Model 1: adiposity ~ ilr(comp) + sq(ilr(comp)) + ses + sex + age + puberty
Model 2: adiposity ~ ilr(comp) + ses + sex + age + puberty
Largest model fitted by lmrob(), i.e. SM

  pseudoDf Test.Stat Df Pr(>chisq)
1     1168
2     1174    9.3493  6     0.1549
```

Quadratic terms were indicated for the fitness composite outcome ($p<0.001$).

```
> anova(mod4, modsq)
Robust Wald Test Table

Model 1: fitness ~ ilr(comp) + sq(ilr(comp)) + ses + sex + age + puberty + bmi
Model 2: fitness ~ ilr(comp) + ses + sex + age + puberty + bmi
Largest model fitted by lmrob(), i.e. SM

  pseudoDf Test.Stat Df Pr(>chisq)
1     1122
2     1128    25.198  6  0.0003138 ***
---
Signif. codes:  0 '***' 0.001 '**' 0.01 '*' 0.05 '.' 0.1 ' ' 1
```

The R function poly(*x*, 2) from the stats package was used to create quadratic terms in the final predictive model because it enables us to get an overall multiple correlation coefficient and p-value for the time-use composition (F=17.0, p<0.001).

```
> mod=lmrob(fitness~poly(ilr(comp),2) +ses+ sex +age + puberty + bmi,control=a1)
> (aov.fit4=car::Anova(mod, test.statistic="F"))
Analysis of Deviance Table (Type II tests)

Response: fitness
                      Df       F    Pr(>F)
poly(ilr(comp), 2)     9  17.037 < 2.2e-16 ***
ses                    1  17.464 3.156e-05 ***
sex                    1  43.973 5.164e-11 ***
age                    1  10.195  0.001448 **
puberty                1   8.701  0.003246 **
bmi                    1 209.199 < 2.2e-16 ***
Residuals           1122
---
Signif. codes:  0 '***' 0.001 '**' 0.01 '*' 0.05 '.' 0.1 ' ' 1
```

Once the best fitting model was determined, this model was used to predict the outcome for a large range of time-use compositions.

2. Prediction of outcomes using regression models

The underlying concept is to use the above regression models to predict the outcome for every possible daily time-use composition, and then to rank the predicted outcomes from worst to best. We restricted the range of possible daily time-use compositions to be within those actually practiced by the participants, and further restricted the ranges of individual behaviours to be within 3SD of their mean value to avoid areas of poor representation of empirical data (i.e., potentially implausible values).

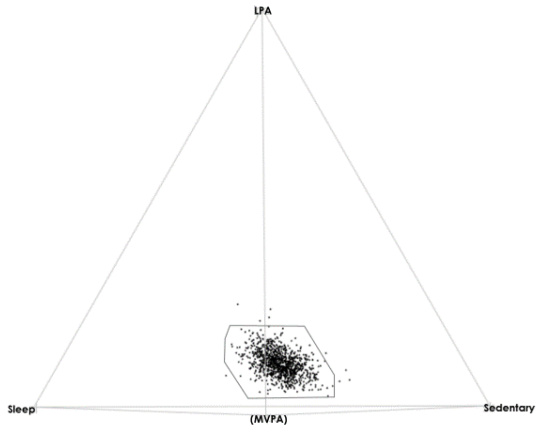

Figure 1 shows the time-use compositions of the included participants (black dots). The black line shape shows the range of the predictive time-use compositions (within 3 SD of the mean of each activity behaviour).

Figure 1. Observed time-use compositions of participants. Apices of triangles represent 100% of each behaviour.

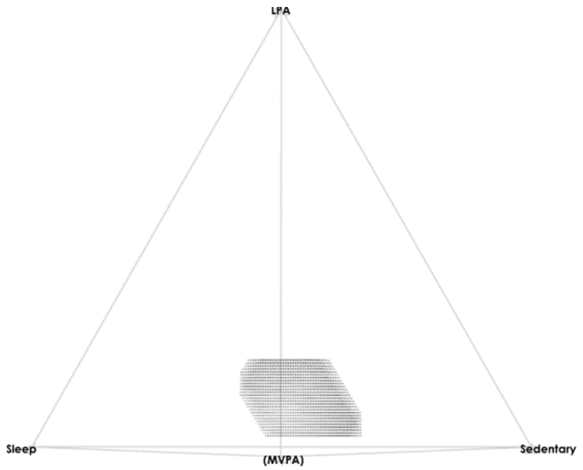

A set of predictive time-use compositions was created within this range, forming a "predictive grid", as shown in Figure 2. There are 6330 datapoints, with 10 minutes between each datapoint.

Figure 2. Predictive grid of time-use compositions

The regression models were used to estimate fitness and adiposity for the predictive grid of time-use compositions in Figure 2, with continuous covariates kept constant at their mean values. Predictions were made for sex=male and sex=female, and then averaged to provide one estimate per time-use composition.

The first six fitness estimates are shown in Table 1 below. There are 6330 rows.

Table 1. Predicted outcome (fitness *z*-score) for time-use compositions (first six).

Sleep	Sedentary	LPA	MVPA	Predicted_Outcome_Zscore
700	690	40	10	-1.403514
690	700	40	10	-1.376661
680	710	40	10	-1.351292
670	720	40	10	-1.327400
660	730	40	10	-1.304984
650	740	40	10	-1.284044

3. Shapes of relationships

The shapes of relationships between individual activity behaviours (e.g., Sleep) and the predicted outcome are visualised by plotting each column in the above Table 1 against the predicted outcome *z*-score (Figure 3).

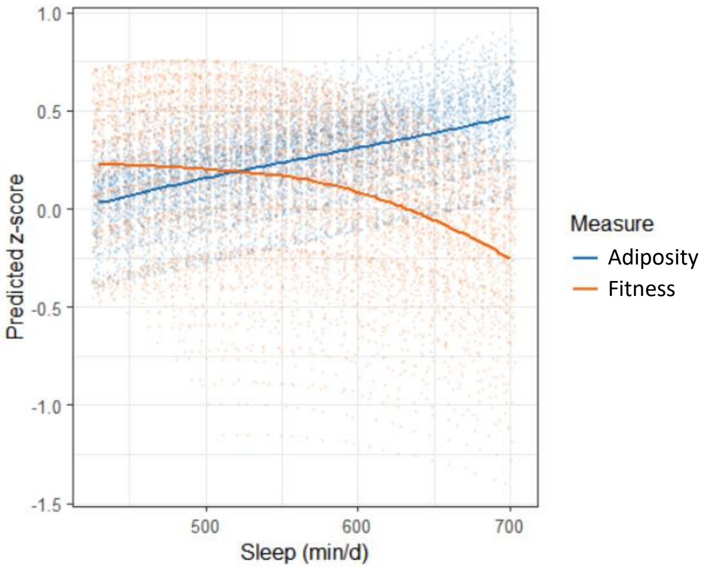

Figure 3. Shape of relationship between sleep and outcomes

4. Best time-use compositions

The predicted outcomes (Table 1) were sorted from worst to best, and the time-use compositions corresponding to the best 5% (the best fitness and adiposity zones) were plotted in Figure 4A below (fitness = orange, adiposity = blue). The best fitness and adiposity zones at 5% did not overlap (Figure 4A). There was overlap of the best fitness and adiposity zones when the best 15% were plotted (Figure 4B, overlap = red). The centre of the overlapping zone (compositional mean) was described as the overall optimal time-use composition for fitness and adiposity.

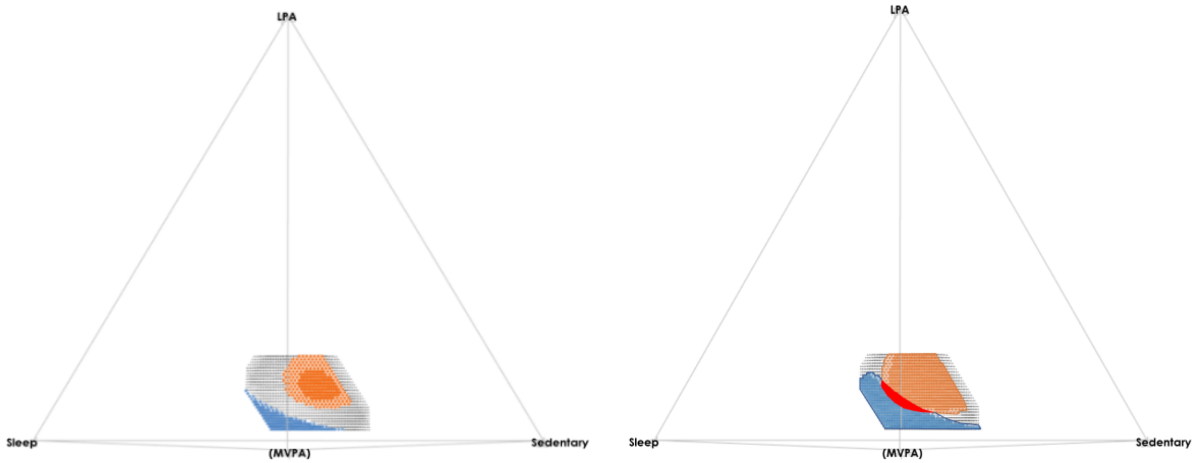

Figure 4. Best fitness and adiposity zones. Left Panel (4A) shows the best 5%, Right Panel (4B) shows the best 15%. Orange=Fitness, Blue=Adiposity, Red=overlapping zone, Black=predictive time-use composition (empirical time-use footprint).
