# Supplementary material for: Balancing time use for children’s fitness and adiposity: Evidence to inform 24-hour guidelines for sleep, sedentary time and physical activity
Source: PLoS One. 2021 Jan 19;16(1):e0245501. doi: 10.1371/journal.pone.0245501 (PMC7815105; doi:10.1371/journal.pone.0245501)
Supplement: S2 File — (PDF) [file pone.0245501.s002.pdf]

S2 File. **Results for Individual Fitness and Adiposity Measures**

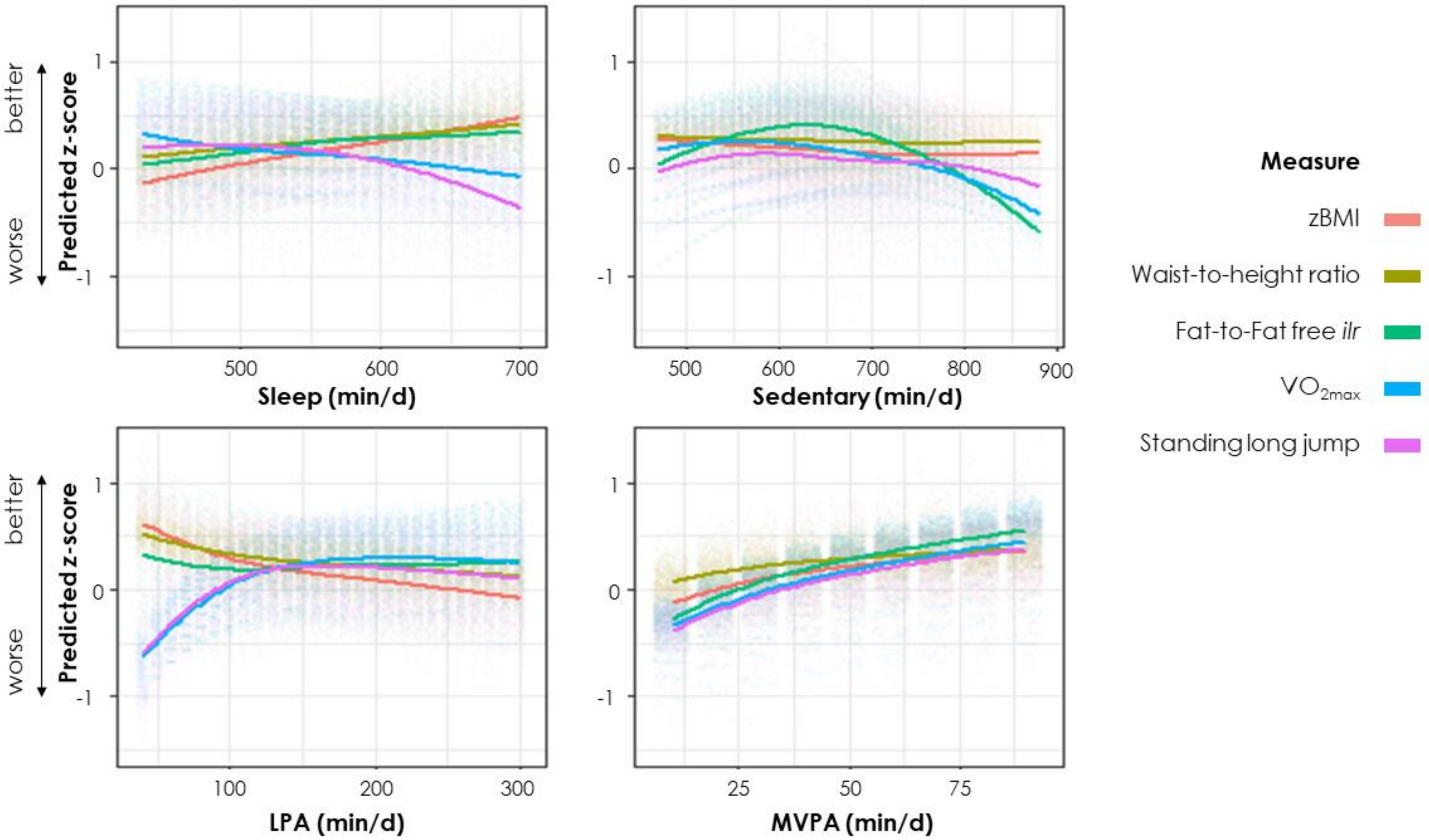

Figure 1. Relationship between incrementally increasing durations of individual activity behaviours and fitness/adiposity outcomes *z*-scores.

Higher *z*-scores represent better outcomes. LPA=light physical activity; MVPA=moderate-to-vigorous physical activity; *z*BMI=body mass index *z*-score; *ilr*=isometric log-ratio; VO_2max=predicted maximal aerobic power. Adjusted for age, sex, puberty and socioeconomic position. Fitness measures additionally adjusted for *z*BMI. Quadratic terms for the composition were included in the body composition (fat-to-fat free *ilr*), VO_{2max} and Jump models. The activity composition was associated with all outcomes (p≤0.001 all models). Note, Jitter was applied to data points to enable visualization of overlapping points. Each data point represents one of the possible permutations of activity compositions (in 10-minute increments) within the study sample's empirical activity footprint (i.e., the ranges of activity durations observed in the sample).
