# Supplementary material for: Balancing time use for children’s fitness and adiposity: Evidence to inform 24-hour guidelines for sleep, sedentary time and physical activity
Source: PLoS One. 2021 Jan 19;16(1):e0245501. doi: 10.1371/journal.pone.0245501 (PMC7815105; doi:10.1371/journal.pone.0245501)
Supplement: Attachment — Submitted filename: Response to reviewers.docx [file pone.0245501.s004.docx]

| **Comment** | **Response** | **Change to manuscript** |
| --- | --- | --- |
| ***Editor*** |  |  |
| Firstly, as mentioned by reviewer two the association between fitness (i.e. VO2) and fatness (BMI) is by default strongly associated. This is because both of them carry identical information, namely weight (Kg), VO2 is expressed as mlO2/kg and BMI as kg/m2. In addition the association between height (m) and O2 are also quite high (reports of r~0.7-0.8). Thus it can be said that those variables are providing almost identical information into the models thus causing collinearity which hampers the interpretation of the models. | Thank you for raising a great point. To clarify, we believe collinearity between VO2max and zBMI is not a concern with the models used in our paper because these two variables are never both considered together as independent variables. The model that includes both VO2 and zBMI has VO2 as the dependent variable, and zBMI as an independent (covariate) variable along with the activity composition. The potential correlations between zBMI and fitness outcomes (and activity composition) is precisely why we included zBMI as a covariate in the fitness models. We wanted to partial out the associations with adiposity.  However, we agree that the potential impact on interpretation of results should be addressed in the Discussion. Please see additional text.  Out of interest we re-ran the analyses without adjusting the fitness model for zBMI. The optimal zone for the fitness composite and the overall best zone only changed slightly, and the overall message remains the same.  Best fitness zone:  unadjusted for zBMI vs adjusted for zBMI  Sleep: 8.3 [7.2; 9.8] vs 8.3 [7.2; 9.7]  Sedentary: 10.6 [9.0; 12.3] vs 10.9 [9.2; 12.8]  LPA: 3.6 [2.3; 5.0] vs 3.4 [2,2; 5.0]  MVPA 1.4 [1.3; 1.5] vs 1.5 [1.3; 1.5]  And the overall best zone (fitness and adiposity):  Fitness model unadjusted for zBMI vs adjusted for zBMI  Sleep: 10.3 [9.3; 10.8] vs 10.2 [9.5; 10.5]  Sedentary: 10.0 [8.7; 11.5] vs 9.9 [8.8; 11.2]  LPA: 2.2 [1.7; 3.3] vs 2.4 [1.8; 3.2]  MVPA: 1.5 [1.3; 1.5]vs1.5 [1.5; 1.5] | In Discussion (Limitations): page 19, lines 363-365.  “We adjusted fitness models for zBMI to partial out potentially confounding adiposity associations. However, conceptually it may not make sense to separate fitness from adiposity, especially because body mass is included in the derivation of VO_2max_.” |
| Secondly, there are a lot of information avaliable regarding the missuse of compositional data analysis and associated methods when analysing accelerometer data and a discussion regarding how you avoided the pitfalls as outlined in Aadland E, Kvalheim OM, Anderssen SA, Resaland GK, Andersen LB. Multicollinear physical activity accelerometry data and associations to cardiometabolic health: challenges, pitfalls, and potential solutions. Int J Behav Nutr Phys Act. 2019 Aug 27;16(1):74. is needed. | Thank you for the reference to Aadland et al. We are aware of this paper and have responded to several of its points here <https://www.mdpi.com/1660-4601/17/7/2220>. (see Section 5.2 on multicollinearity)  Briefly, Aadland et al used a type of log-ratio transformation (centred log ratios) for their correlation analyses which is inappropriate for such analyses due to their inherent perfect multi-collinearity. We have used isometric log-ratios, which are not perfectly multi-collinear. However, it is possible to have high collinearity between isometric log ratios (as with any variables), and this should always be checked (as in any analysis) when constructing models. In our case, the variance inflation factors for the set of log ratios was well below acceptable limits (highest for log ratios = 1.58, highest for any independent variable =1.7). However, this is largely inconsequential in the analyses we conducted as we do not interpret individual regression coefficients. We use our models for prediction. Even with high multi-collinearity between predictor variables, models can be used for prediction provided the new input data does not extrapolate too far from the data used to fit the model (<https://onlinelibrary.wiley.com/doi/abs/10.1002/wics.84>).  Please see additional text in methods regarding variance inflation factors. | Methods: page 10, lines 207-208.  “Variance inflation factors for exposure variables were below acceptable limits in all models (all < 1.7) (39).” |
| Please ensure that your manuscript meets PLOS ONE's style requirements, including those for file naming. | We have named files appropriately and checked style requirements. |  |
| In your Methods section, please provide additional details regarding the recruitment of participants to this study, including any inclusion and exclusion criteria | Additional details have been provided as requested. | Methods: page 6, lines 108-111:  “All children in LSAC Wave 6 (n=3764) were invited to participate in CheckPoint, without exclusion. A total of 1874 participated in CheckPoint, and of these, 1279 had valid and complete accelerometry data. Participants with complete accelerometry, outcome and covariate data were included in this study (n=1182 for adiposity and n=1137 for fitness).” |
| We note that you have indicated that data from this study are available upon request. PLOS only allows data to be available upon request if there are legal or ethical restrictions on sharing data publicly. For information on unacceptable data access restrictions, please see [http://journals.plos.org/plosone/s/data-availability#loc-unacceptable-data-access-restrictions](https://protect-au.mimecast.com/s/9UdJC0YK3oIMvWZySO2Xfb?domain=journals.plos.org). | Unfortunately there are legal restrictions on sharing the data publicly. To become a licenced data user for the CheckPoint and LSAC studies researchers are required to sign a confidentiality deed [https://ada.edu.au/wp-content/uploads/2019/11/Confidentiality-Deed-Poll-November-2019.pdf](https://protect-au.mimecast.com/s/q_h1CwV1mRCoE4xmtVtgHs?domain=ada.edu.au). The confidentiality deed imposes a legal obligation to not share any unit-level data with anyone unless they also are registered data users. Researchers can apply to become registered data users by following the link provided below. https://growingupinaustralia.gov.au/data-and-documentation/accessing-lsac-data. |  |
|  |  |  |
| Reviewer 1 |  |  |
| Assessment of physical activity and the use of accelerometry data. The Authors present amounts of LPA and MVPA in relation to adiposity and fitness outcomes. I think it is important that the Authors consider that any fixed intensity cut-off would cause significant miss-classification of PA intensity. This is especially important when one outcome is physical fitness. It is plausible that children with better fitness levels achieve those fixed thresholds with less effort than lower with children and therefore underestimate their time spent in MVPA (e.g. PMID: 32620888). This is crucial point as miss-classification PA intensity would cause remarkable bias to the results. In addition, the PA measure may be a source of bias because the validation study used to create the used the intensity cut-offs utilised MET-values in creating the cut-offs. METs are inherently confounded by adiposity (PMID: 26321958) and fixed MET-based cut-offs cause misclassification of PA intensity (see PMID: 32620888). Of note, MET cut-offs 3-4 and 6-7 are often used, but they lack reasonable physiological rationale. Furthermore, in addition to possible misclassification of PA intensity, the results would markedly differ in any other accelerometry cut-points would have been used (PMID: 30548545). I acknowledge that currently there are very limited possibilities for individual calibration of accelerometry cut-points, but keeping this information in mind I suggest that the authors more carefully interpret the results and include some discussion about this perhaps into limitations section. | We agree with the reviewer and thank them for the interesting references. Unfortunately using fixed intensity cut-offs to classify activities may cause miss-classifications. We have addressed this as a limitation in the Discussion. | Discussion (limitation): page 19 line 369- page 20 line 377.  “Particularly, the use of fixed intensity cut-offs to classify intensity of physical activity may have introduced bias, especially for fitness models. This is because children with better fitness may achieve fixed thresholds with less effort than children with poorer fitness, potentially underestimating their time in MVPA (48). In addition, the validation study used to create the cut-offs used metabolic equivalents (METs) which are inherently confounded by adiposity (49). Thus, different decisions made during the accelerometery processing may lead to different daily activity estimates and different predicted optimal activity durations. Research using other datasets, cut-points and accelerometers is encouraged to validate or challenge our findings.” |
| Can the authors provide some reason for 60 s epoch? It is rather long for paediatric studies. | The accelerometry data provided in the CheckPoint data release are already classified into energy expenditure bands using 60-second epochs, so we are unable to change this. We have added a sentence to the Discussion explaining how the relatively long epoch may affect estimates of MVPA | Discussion (limitation): page 19, lines 367 – 369.  “Accelerometer counts were collapsed into 60-second epochs, which is long in comparison to other paediatric studies, and may have contributed to relatively low estimates for MVPA in this study.” |
| Vigorous intensity PA has been considered important for health outcomes and based on evidence for intervention studies it would be more important for cardiorespiratory fitness than moderate intensity exercise. VPA is also included the PA recommendations by WHO and PA recommendations for Americans. Thus, I suggest that the Authors also include VPA into the manuscript | We agree that VPA is important, however, we prefer to keep the 4-part composition (sleep, sedentary, LPA, MVPA) for this study as it is possible to visualise and it is relevant to current 24-hour guidelines around the world, which this study speaks to. Current recommendations around VPA are quite generic (e.g., should be incorporated three times a week), so including durations for VPA in daily activity guidelines will require some re-conceptualization of current 24-h guidelines. We acknowledge the reviewer’s point and have added the exploration of VPA as an avenue for future research. | Discussion: Future directions: Page 22, lines 415 – 420.  “Our study collapsed moderate and vigorous intensities of physical activity into MVPA because MVPA is relevant to current 24-hour movement behavior guidelines. However, some studies have found differential health associations for moderate and vigorous physical activity, for example, vigorous physical activity appears more beneficially associated with cardiorespiratory fitness than moderate physical activity (51). It would be of interest for future studies to explore the two intensities separately.” |
| I also suggest that the Authors interpret the cardiorespiratory fitness data carefully as VO2peak normalised for body mass is not a pure measure of aerobic power but also adiposity making it more a measure of functional capacity than physiological measure of the function of cardiovascular and skeletal muscle systems (please see e.g. reviews by Welsman and Armsntrong). Thus, I suggest that the Authors also highlight this limitation and discuss how it may affect on the results | Thank you for bringing this to our attention. We attempted to partial out the associations of adiposity from the fitness models by adjusting for zBMI. We now highlight this as a limitation and discuss how it may affect our results. (see response to editor) | In Discussion (Limitations): page 19, lines 361-365.  “It should be considered that VO2max was normalized for body mass, meaning it was more a measure of functional capacity rather than a measure of aerobic power. We adjusted fitness models for zBMI to partial out potentially confounding adiposity associations. However, conceptually it may not make sense to separate fitness from adiposity, especially because body mass is included in the derivation of VO2max.” |
| The introduction would benefit more detailed description why each component (incl. VPA) would be associated with adiposity and fitness. | A more detailed description has been added to the introduction. | Introduction. Page 4, lines 56 – 64.  “Fitness is likely to improve when more time is spent in activities of higher intensity (moderate and vigorous), which load the cardiorespiratory system and provide opportunity to build muscle strength and power (1). Conversely, fitness may decline when less time is spent in such activities, in favor of sedentary behaviors and sleep. Similarly, adiposity may decrease when children spend more time in moderate- and vigorous-intensity activities because their energy expenditure increases. Getting sufficient sleep may also lead to decreased adiposity due to better regulation of stress hormones which conserve energy (2). It may be possible that too much sedentary and light activity could rob time from higher intensity activities, increasing adiposity.” |
| L99. How sleep was derived from the accelerometry data? I assume that sleep was treated as more is better? Please also consider that more is not always better | Further detail about sleep derivation has been added to methods.  We agree that more is not always better.  We made no assumptions about more or less sleep being better. We included quadratic terms to the activity log ratios where indicated (fitness models) to allow for non-linear relationships. | Methods: page 7, lines 132-137.  “A MATLAB-based customized software program (Cobra), the algorithm by van Hees et al (25), visual inspection of accelerometer traces and the paper-based logs were used to identify sleep and non-wear time. The 60-second epochs were classified as sleep if they contained equal or more sleep vs wake 5-second epochs.” |
| Assessment of body composition. Were bioimpedance analysis taken in fasted stated or how was it standardised? Please explain. Please also explain why body composition was expressed at it was. To enhance the comparability between studies the Authors could also report BF% in the supplement. Does composite adiposity score add anything to this paper. What the Authors think that it describes? | BIA measures were taken in semi-fasted condition (median about 4 hours). This information has been added. Variation in timing since breakfast may have induced small variation in body composition.  Body composition was expressed as the isometric log ratio between body fat and fat-free mass because body composition is a compositional variable (see recent statistical paper <https://journals.sagepub.com/doi/10.1177/0962280220955221>  )  We now report %BF in the descriptive table.  Composite scores were used for adiposity and fitness to provide a summary of the overall construct from the available variables. The adiposity composite incorporates a selection of slightly different aspects of adiposity, including waist circumference as well as body composition and weight status (standardised zBMI). | Methods: page 9, line 167.  Body composition and weight were measured in semi-fasted condition,”  Limitations: page 19, lines 359-361.  “Body composition was measured in a semi-fasted state, thus small differences may have been due to timing of the participant’s latest meal.”  Methods: page 9, line 172.  “Body composition was expressed as the isometric log ratio (ilr) of fat vs fat-free mass, 1/√2 ((Fat mass)/(Fat-free mass)) to respect the compositional nature of the variable (32).”  Table 1 now includes descriptive summary of %BF |
| Please adjust the discussion according to previous comments | We have adjusted the discussion as detailed in our previous responses. | See red-coloured text in revised Discussion |
| Please be careful with any causal terminology as this was cross-sectional study. | We were very careful remove cause and effect language. We agree that the term “optimal” may have causal implications. Thus, we have clarified this in our Discussion. | Limitations: page 19, lines 355-357.  “Limitations include the cross-sectional design, precluding inference of causation or directionality. Although we talk about optimal days and durations, we cannot imply that achieving these activity durations will bring about improvements in outcomes.” |
| L270. Please be careful in expressing volume number true because thresholds and methods used to assess PA will have effect on those estimates. | We agree, and have added a qualifying statement. | Page 17, lines 316-317.  “Based on the activity classifications used in this study, the best trade-off day to optimize both outcomes was estimated to be 10.2 h sleep, 9.9 h sedentary time, 2.4 h LPA and 1.5 h MVPA.” |
|  |  |  |
| Reviewer 2 |  |  |
| Line 63 and 65: The wording adiposity seems quite confusing in combination with 'better fitness' or 'beneficial'. Please use lower weight status or similar. | The terminology for adiposity or fatness is indeed tricky because the “good” direction is opposite to that for fitness. We tried a selection of different terms but decided on adiposity because it speaks to all three indicators in our composite variable: waist circumference, body composition (%BF vs FFM) and weight status (zBMI). We feel the suggested “weight status” term does not necessarily cover waist circumference and results in challenges with interpretation in terms of directionality (i.e., is an increase in weight status good or bad?). Thus, we would prefer to retain the adiposity wording. | No change. |
| Line 76f.: The authors might include more information on prior work about compositional data analysis. | Thank you for this suggestion. We have included more about this in the introduction. | Introduction, page 5, lines 92-97:  “An increasing number of CoDA studies are exploring the relationships between daily activities and health outcomes (12). In general, these studies have identified that higher MVPA, relative to the other daily activities, is beneficially associated with both children’s fitness and adiposity, while longer sleep, shorter sedentary time and shorter LPA (each relative to other activities) is associated with lower adiposity only (13-17).” |
| Line 85-90: Some information about the sample size from Fig 1 might be included in the text. | Agree, this has been added. | Methods: page 6, lines 108-111.  “All children in LSAC Wave 6 (n=3764) were invited to participate in CheckPoint, without exclusion. A total of 1874 participated in CheckPoint, and of these, 1279 had valid and complete accelerometry data. Participants with complete accelerometry, outcome and covariate data were included in this study (n=1182 for adiposity and n=1137 for fitness).” |
| Line 106: A short description of the algorithms (at least concept and main parameters) for sleep detection and non-wear time (similar to non-wear detection by Choi?) should be included for the reader. | Agree, this has been added | Methods: page 7, lines 132-135.  “A MATLAB-based customized software program (Cobra), the algorithm by van Hees et al (25), visual inspection of accelerometer traces and the paper-based logs were used to identify sleep and non-wear time. The 60-second epochs were classified as sleep if they contained equal or more sleep vs wake 5-second epochs.” |
| Line 172: Please state the covariates that are used for adjustment. | Statement has been added. | Methods: page 9, lines 184-185.  “Models were adjusted for sex, age, pubertal development and socioeconomic position. Fitness models were additionally adjusted for zBMI.” |
| Line 215: Again the use of adiposity. Best mean adiposity should be actually a mean weight status, right? high weight status refers to adiposity but optimal adiposity seems to be average weight status? Please correct precisely | We were unclear as to what the reviewer has requested. We think this was addressed in our response to the reviewer’s first point about the use of “adiposity”?  When talking about adiposity we refer to the composite score which includes three separate measures. | No change. |
| Line 239: Please discuss the use of the term adiposity measure, since you actually use weight status | The variables included in the adiposity composite are listed in the legend of the Table. (Adiposity composite includes zBMI, waist-to-height ratio and fat-to-fat-free mass *ilr*). They are not referring to weight status. | No change. |
| Line 327: The term lower adiposity actually should refer to zBMI, i.e. weight status. Please reconsider the wording. | Lower adiposity refers to the overall construct of adiposity – it includes three separate indicators each covering a slightly different aspect of adiposity, not weight status per se. | No change. |
